# Robust acoustic directional sensing enabled by synergy between resonator-based sensor and deep learning

**DOI:** 10.1038/s41598-024-60696-1

**Published:** 2024-05-02

**Authors:** Ziqi Yu, Xiaopeng Li, Hojung Jung, Masahiro Harada, Danil Prokhorov, Taehwa Lee

**Affiliations:** 1 Toyota Research Institute of North America, Toyota Motor North America, Ann Arbor, MI 48105 USA; 2 Toyota Motor Corporation, 1200 Mishuku, Susono, Shizuoka 410-1107 Japan

**Keywords:** Engineering, Physics

## Abstract

We demonstrate enhanced acoustic sensing arising from the synergy between resonator-based acoustic sensor and deep learning. We numerically verify that both vibration amplitude and phase are enhanced and preserved at and off the resonance in our compact acoustic sensor housing three cavities. In addition, we experimentally measure the response of our sensor to single-frequency and siren signals, based on which we train convolutional neural networks (CNNs). We observe that the CNN trained by using both amplitude and phase features achieve the best accuracy on predicting the incident direction of both types of signals. This is even though the signals are broadband and affected by noise thought to be difficult for resonators. We attribute the improvement to a complementary effect between the two features enabled by the combination of resonant effect and deep learning. This observation is further supported by comparing to the CNNs trained by the features extracted from signals measured on reference sensor without resonators, whose performances fall far behind. Our results suggest the advantage of this synergetic approach to enhance the sensing performance of compact acoustic sensors on both narrow- and broad-band signals, which paves the way for the development of advanced sensing technology that has potential applications in autonomous driving systems to detect emergency vehicles.

## Introduction

Evolution has enabled the creatures of the ability to acoustically sense the surrounding environment for information on the location of approaching objects, which is critical for their survival. The acoustic sensors with similar capability are likely important for humans as approaching to a more intelligently connected world in applications such as detection, navigation, and communication^1–4^. Conventional directional sound sensors rely on processing different time of arrival of spatially apart detectors, which is challenging in compact subwavelength systems^5–7^. Bio-inspired MEMS directional sensors, which were based on coupled resonating structures that were sensitive to incoming angles of sounds, have proven promising performance in subwavelength designs, but still suffered from limited sensing range and fabrication challenges^8–11^. Such a sensing range limit has been overcome by MEMS directional microphone array demonstrating sound source localization in three dimensions^12^, yet requiring sophisticated signal processing equipment (e.g., lock-in amplifiers). Alternatively, various other types of miniaturized realization of sound directional sensors have been implemented based on metamaterials^13,^^14^, and metamaterial enclosure^15^, which were limited by narrow sensing range and pre-requisite of additional information. Recently, a subwavelength sound directional sensor based on three radiatively-coupled resonators were demonstrated and showed 0°–360° sensing capability, which was enabled by incident angle dependent ratio of acoustic energy in the resonators^16^. The amplitude of the incoming sound was significantly enhanced by the resonators while the increased phase difference between microphones was not as much to be processed for directional sensing.

The advancement of neural networks has enabled their implementation in various sound-related applications including the estimation of direction of arrival^17–19^, sound source localization^20–28^, speech recognition^29^, voice activity detection^30^, and even multi-tasking^31–33^. Network architectures gradually evolved from shallow perception models^20^ to convolutional neural networks^18,21^ and their combination with recurrent neural networks^31^, attention-based networks^25^, encoder-decoders^24^, and U-Net^26^. The increasingly complex networks and added components have enabled improved acoustic sensing performance on single^30^, multiple^24,25^, and moving^26,33^ sources in reverberant and noisy environments that are closer to real-world application scenarios. The detection and localization of the sound source have been treated as either classification^17,27^ or regression^22,28,32^ by using neural networks with input features derived from sound magnitude^23^, phase^18,34^, or their combination^19,33^. For detailed discussion on the development of using neural networks for acoustic sensing, we refer the readers to a recently published comprehensive review^35^.

While existing work has demonstrated the versatility of neural networks in a variety of acoustic sensing applications, however, most focused on refining learning algorithms, feature extractions, and network architectures. Little attention has been paid to the understanding of physical systems that directly interact with signals and the underlying physics behind the extraction of acoustic features. Recent studies^36,37^ have shown improved neural network performance thanks to considerable changes in features in the presence of metamaterials. Resonators are well known for amplifying the acoustic response near the resonance. Whether the enhanced acoustic features could be effectively harnessed by neural networks for more accurate and robust acoustic sensing remains an open question.

Aiming at bridging abovementioned gaps, in this work, we fabricated and measured acoustic sensors with and without (reference) resonators on single-frequency and sirens signals and trained CNNs by different acoustic features (i.e., the amplitude, the phase, and both). Through comparing the CNN performance between resonator-based and reference sensors, we show that resonance effect improves the training features for neural networks, significantly enhancing their accuracy on estimating sound source direction especially when amplitude and phase are simultaneously used in the training. For our subwavelength device, it may be intuitive to expect less importance from the phase due to limited spatial separation between detectors. We demonstrate that CNNs effectively differentiate and extract phase features that may be overlooked by conventional methods. For our resonator-based sensor, we consistently observe better accuracies from the CNNs trained using both amplitude and phase extracted from single-frequency (narrowband) and siren (broadband) signals, despite the signals contain noises, which demonstrate a beneficial synergy between resonance effect and deep learning as well as the robustness of this hybrid approach.

## Data preparation and deep learning

### Acoustic sensor and experimental measurement

**Figure 1:**
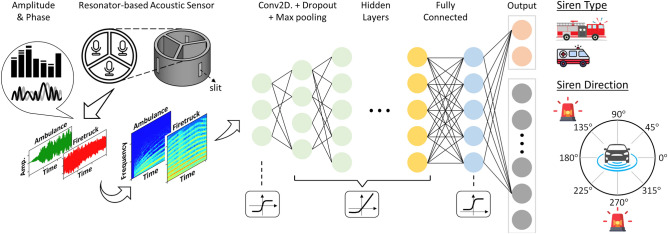
Workflow of the deep-learning assisted acoustic angle sensing of sirens.

The workflow of performing the acoustic sensing by employing CNN is shown in Fig. 1. Our aim is to investigate the use of CNN in combination with acoustic resonators for spatial localization of stationary sound sources. While recurrent neural networks (RNNs) are better suited for tasks that require modeling temporal dependencies, such as predicting the direction of a moving sound source, we believe that CNNs are more effective for our specific purpose in this work as they provide effective separation of the phase and amplitude information as inputs. For this reason, we have chosen CNNs rather than experimenting on various deep learning algorithms.

Time-domain signals are experimentally obtained from our fabricated acoustic sensor. Here, we provide a brief description of the sensor design and measurement setup and further details can be found in^16^. The 3D-printed resonator-based sensor (Markforged Mark Two, Markforged Inc.) possesses three resonant cavities and its diameter, wall thickness, and the height are $d$ = 52 mm, $t$ = 4 mm, and $h$ = 25 mm, respectively, while the slit has a height of $h_{s}$ = 10 mm and width of $w_{s}$ = 2 mm, respectively. In our experiments, three surface microphones (130B40, PCB Piezotronics) are positioned inside the cavities and a sound source is placed at different angles in the xy-plane with respect to the sensor to produce the incoming sound field. Different incident angles (we have chosen from 0 to 350° by every 10^∘^, leading to 36 incident angles) are realized by a motorized rotation stage (PRMTZ8, Thorlabs) on which the sensor is mounted while the sound source remains still. The responses of the three microphones are collected for 3 s at a sampling rate of 96 kHz through a data acquisition system (NI USB-4431, National Instruments). The measurements were performed in a study room (11 ft wide by 10 ft long) and the distance from the loudspeaker (5″ diameter) and the acoustic sensor is fixed at 0.3 m. The sensor is carefully positioned on the axis of the loudspeaker to minimize the deviation of sound amplitude due to off-axis positioning. This environment, which includes undesired reflection from surroundings, allows us to evaluate the robustness of our hybrid approach. While we do not consider varying the distance between the source and sensor, previous studies have demonstrated estimating the distance from the sound source^38–41^. More details about the design of the sensor and the experimental measurement can be found in Section S1 of the Supplementary Materials.

### Acoustic sensing via deep learning of single-frequency or siren sources

To extract the amplitude and/or phase feature for deep learning, the time-domain signal (3-s long) is converted into a spectrogram^37,42–44^ through short time Fourier transform (FFT window size of 10.7 ms, hopping of 5.3 ms, and with the amplitude being in dB scale), which is a two-dimensional (2D) time–frequency representation, whose real and imaginary components yield amplitude and phase. The spectrogram is regarded as an 2D image and is subsequently fed into a CNN, which is known to be good at handling image recognition tasks. For three microphones, the CNN input data consists of 6 channels associated with the real and imaginary components from each of the 3 resonators. When the network is trained by both features, all 6 channels are used, whereas the training by either amplitude or phase feature utilizes the corresponding 3 channels. In the CNN, as shown in Fig. 1, each 2D convolutional layer is followed by a dropout and a max pooling layer to prevent overfitting. L_2_ normalization is implemented for the same purpose. The input layer is activated by the hyperbolic tangent (tanh) function, while the subsequent layers are instead activated by the exponential linear unit function. The CNN prediction of source direction angle is regarded as a classification task with 36 classes^17,45,46^ corresponding to 36 angles consistent with experiments (10° internal in 0–350° range). The CNN predicts an angle among these as the output. More details about the CNN architecture can be found in Fig. S2 and Section S2 of the Supplementary Materials.

For the siren sound, the CNN predicts both the incident angle and the type of the siren. In this work, we have used the sirens of ambulance and firetruck pre-recorded and available from open-source data^47^. Each recorded file contains only one type of siren (so that we do not tackle multiple sirens that are simultaneously active), with a variety of background noises such as running cars nearby, horns, bus stops, and pedestrian talking, etc. We have carefully inspected the siren quality (i.e., reasonably clear and does not sound too distant) and have chosen 18 recordings (8 and 10 for ambulances and firetrucks, respectively) for the experiment, in which for each incident angle the measurement is repeated 6 times for a siren. With the total 36 incident angles, we end up preparing 3888 samples, which are split at random, by 80%, 10%, and 10% into the training, validation, and testing datasets, respectively. The CNN trained by datasets split this way works well and hence, throughout this work, we skip additional k-fold validation. The output layer of the network that predicts the angle and type is activated by the softmax function with 36 and 2 classes, and the respective loss functions adopted in the Adam optimizer are categorical cross entropy and the binary cross entropy, which are assigned equal weights. Throughout this paper, when quantifying the performance of the trained CNN, we have defined the validation and test accuracy both as an exact match between the prediction and the ground truth, though, in the literature, a tolerance range has also been used^46^.

While it is intuitive to expect our sensor to work better around the resonance, we will show later that the combination with the CNN enables good performance for siren sources, which cover wide range of off-resonant frequencies. As a complement, we measure the sensor’s response to single-frequency signals following abovementioned procedure while repeating the measurement for each incident angle 4 times, yielding 3744 samples (i.e., 4 × 26 frequencies × 36 angles). In this case, the CNN only predicts the incident angles unlike both the angles and the types for the siren. We also take one step further by including the scenario where two sources are active simultaneously. To this end for simplicity, we synthesize the data by combining the previously-measured single source responses at two incident angles drawn from the total of $36n$ possible pairs, which can be expressed by the combination ${\text{C}}\left( {36n,2} \right)$ with $n$ being the number of frequencies. This eliminates the necessity of experimentally exhausting all possibilities of incident angles over the considered frequency range, which would be prohibitively time-consuming.

## Results and discussion

### Resonant characteristics of the acoustic sensor

**Figure 2:**
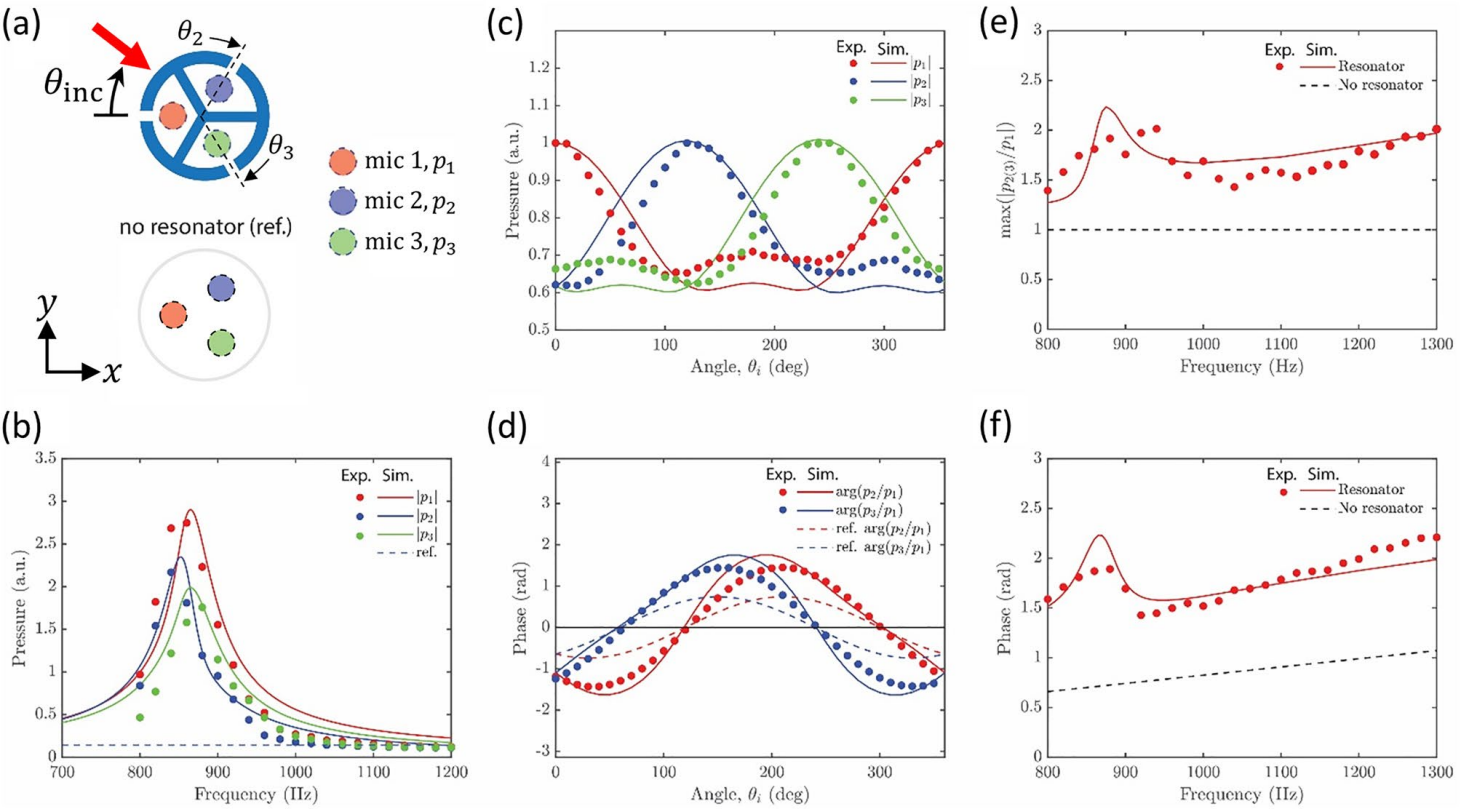
(**a**) Schematic of the resonator-based acoustic sensor (top) having three resonant cavities and the acoustic sensor without resonators (bottom, referred to as reference). When measuring the reference sensor, the microphones are inserted into the same positions as those of the resonator sensor for fair comparison. Dashed circles indicate the positions where acoustic pressures are calculated in the simulations. The legends “Exp.” and “Sim.” correspond to the experimental measurements and the FEM simulations. (**b**) Spectra of acoustic pressures in three cavities of the resonator-based sensor when incident angle is $\theta_{inc}$ = 40°. Variations of (**c**) acoustic pressures and (**d**) phases in three cavities at 900 Hz for different incident angles. (**e**) Maximized ratio between acoustic pressures in two cavities and (**f**) phase difference over the range of frequency from 800 to 1300 Hz.

The responses of the acoustic sensor with and without resonators (reference case) are compared by conducting numerical simulations using the commercially available software COMSOL Multiphysics 5.3. The resonator-based sensor is modified to have a lower resonance frequency and to accommodate surface microphones for robust sensing, as indicated by the circles in Fig. 2a [consistent color codes are used in Fig. 2b and c]. The acoustic pressure is calculated inside each cavity when the incident acoustic field comes from different angles ranging from 0 to 350°. The losses are considered by using the narrow region acoustics, which defines a fluid model for viscous and thermal boundary-layer-induced losses occurring in the slits (i.e., the openings of the Helmholtz resonators). For a fair comparison, the acoustic pressure of the sensor without resonant cavities [“no resonator (ref.)” case as depicted in Fig. 2a] is computed at the three locations matching those of the microphones in the resonator-based sensor. In Fig. 2b, we compare the acoustic pressures inside three cavities, namely, $p_{1}$, $p_{2}$, and $p_{3}$, by showing the vibration amplitudes $\left| {x_{1} } \right|$, $\left| {x_{2} } \right|$, and $\left| {x_{3} } \right|$ since $p_{j} = - p_{A} \gamma_{s} S_{j} x_{j} /V_{j} \left( {j = 1,2,3} \right)$ with $p_{A}$, $\gamma_{s}$, $S_{j}$, and $V_{j}$ being the atmospheric pressure, the ratio of the specific heat (1.4 for air), the cross-section area of the *j*th slit, and the volume of the *j*th cavity. We have chosen the incident angle of $\theta_{inc}$ = 40° as an example. We can see that over the range of frequency from 700 to 1200 Hz, three well-defined resonant peaks appear at a frequency around 900 Hz, which corresponds to the designed resonant frequency of the sensor. Notably, the peak magnitudes differ due to the positions of resonant cavities relative to the incident acoustic field. Particularly, in Fig. 2b, the incident sound hits the cavity 1 and then reaches cavities 2 and 3, whose amplitudes slightly reduce because of acoustic shadowing effect. Interestingly, the resonators do not work as filters, which remove acoustic features away from resonant frequency. Instead, even at 1200 Hz, the amplitudes for these frequencies approach the reference values, which are represented by a dashed horizontal line in Fig. 2b. Experimental results are shown by symbols at an interval of 20 Hz, which achieve a fair agreement with the simulation data. In Fig. 2c, variations of acoustic pressures at 900 Hz with respect to incident angle $\theta_{inc}$ are illustrated with an interval of 10°. We note that for each $\theta_{inc}$ a unique combination of $\left( {\left| {x_{1} } \right|, \left| {x_{2} } \right|, \left| {x_{3} } \right|} \right)$ can be found for the sensor with the resonators, whereas for the reference case, no variable appears. The experimental results agree with the trend of the simulations with slight mismatches. We note that unlike our previous work^16^ which performed the measurement in a well-controlled environment (2D waveguide) and used anechoic foams to minimize reflection from the surrounding, the current measurements were conducted in a study-room environment without any countermeasure to undesired reflections. Additionally, only the sensor was exposed to the incoming sound waves in previous experiments^16^; however, the current setup also includes reflections from the fixtures (e.g., rotation stage) and possible misalignments between sound source and the sensor. Despite a high signal-to-noise ratio (SNR) of 30 dB [Fig. S3a of the Supplementary Materials], which indicates room quietness, SNR does not measure unwanted reflections and misalignment, which could be a potential reason for the discrepancy observed between our previous study and Fig. 2c in this work. Similarly, in Fig. 2d, the phase in each cavity with respect to $\theta_{inc}$ is displayed. Non-intuitively, the phases of incident sound after passing through the resonators are also enhanced in comparison with the reference case, though not as significant as the amplitudes. This simulation observation is further confirmed by the experimental data, showing good agreements in both the trend and magnitude. Figure 2c and d clearly demonstrate the advantage of resonator-based acoustic sensors in preserving and enhancing useful amplitude and phase features, which will benefit angle sensing performance as we will see later.

To further understand the resonant enhancement of both amplitude and phase, we look into an analytical model, which is given by ^48,49^

1 $$m_{j} \frac{{d^{2} x_{j} }}{{dt^{2} }} + \left( {{\Gamma }_{jj} + {\Delta }_{j} } \right)\frac{{dx_{j} }}{dt} + k_{j} x_{j} + \mathop \sum \limits_{n \ne j}^{N} {\Gamma }_{nj} \frac{{dx_{n} }}{dt} = F_{j} ,$$

where $x_{j}$ is the displacement of the mass ($m_{j}$) of the $j{\text{th}}$ resonator, ${\Delta }_{j}$ is the loss [kg/s], $k_{j}$ is the spring constant, ${\Gamma }_{j0}$ is the leakage [kg/s], ${\Gamma }_{ij}$ is the coupling between $i{\text{th}}$ and $j{\text{th}}$ resonators [kg/s], and $F_{j}$ is the force acting on the $j{\text{th}}$ resonator. Because of the discrete rotation symmetry, Eq. (1) is further simplified with $m_{j} = m$, $\gamma_{0} = {\Gamma }_{jj} /m_{j}$, $\delta = {\Delta }_{j} /m_{j}$, $\gamma = {\Gamma }_{ij} /m_{j}$, $k = k_{j}$, and $\omega_{0}^{2} = k_{j} /m_{j}$ ($\omega_{0}$: resonance frequency). The resonators in our acoustic sensor couple with each other via the radiation leakage through the environment. Analytically, it reads ${\Gamma }_{ij} = \left( {i\omega \rho S} \right)/\left( {2\pi k_{w} R} \right)\mathop \sum \limits_{ - \infty }^{\infty } \left( {H_{n} \left( {k_{w} R} \right)/H_{{n}^{\prime}} \left( {k_{w} R} \right)} \right)e^{{in\left( {\theta_{i} - \theta_{j} } \right)}}$, which quantifies the leakage rate between the *i*th and *j*th resonators, where $k_{w}$ and $\omega$ refer to the wavenumber and the angular frequency, $\rho$ is the mass density, $S$ and $R$ denote the slit cross-section area and the cylinder radius, $H_{n}$ and $H_{n}{\prime}$ are the *n*th-order Hankel function and its derivative, and $\theta_{i\left( j \right)}$ is the angle determined by the *i*th resonator position. We note that the damping coefficients $\gamma$, $\gamma_{0}$, and $\delta$ were extracted numerically and have typical values of $\gamma_{0} \approx 0.02\omega_{0}$, $\gamma_{0} \approx 0.025\omega_{0} - 0.06\omega_{0}$, and $\delta \approx 0.1\omega_{0}$, respectively. Alternatively, they can be analytically found. In Fig. S4 of the Supplementary Materials, we provide a comparison between simulation results and analytical results. The analytical results are based on harmonic oscillator model using numerically obtained damping coefficients, which shows good agreement. In the frequency domain, Eq. (1) can be explicitly written for three resonators (*N* = 3) as,

2 $$\left[ {\begin{array}{*{20}l} A \hfill & { - i\omega \gamma } \hfill & { - i\omega \gamma } \hfill \\ { - i\omega \gamma } \hfill & A \hfill & { - i\omega \gamma } \hfill \\ { - i\omega \gamma } \hfill & { - i\omega \gamma } \hfill & A \hfill \\ \end{array} } \right]\left[ {\begin{array}{*{20}c} {x_{1} } \\ {x_{2} } \\ {x_{3} } \\ \end{array} } \right] = \left[ {\begin{array}{*{20}c} {f_{1} } \\ {f_{2} } \\ {f_{3} } \\ \end{array} } \right],$$

where $A = - \omega^{2} + \omega_{0}^{2} - i\omega \left( {\gamma_{0} + \delta } \right)$, and $f_{j}$ is the force acting on the $j{\text{th}}$ resonator, i.e., $f_{j} = F_{j} /m = f_{0} e^{{i\theta_{j} }}$ with $\theta_{j} = \left( {2\pi R/\lambda } \right) \times \cos \left[ {2\pi \left( {j - 1} \right)/3 - \theta_{inc} } \right]$. From Eq. (2), the displacement ratio between resonators 1 and 2 is expressed as

3 $$\frac{{x_{1} }}{{x_{2} }} = \left[ {\frac{{A^{2} + \omega^{2} \gamma^{2} + \left( { - \omega^{2} \gamma^{2} + i\omega \gamma A} \right)\left( {f_{2} + f_{3} } \right)/f_{1} }}{{A^{2} + \omega^{2} \gamma^{2} + \left( { - \omega^{2} \gamma^{2} + i\omega \gamma A} \right)\left( {f_{2} + f_{3} } \right)/f_{2} }}} \right] \times \frac{{f_{1} }}{{f_{2} }} = \alpha e^{i\beta } \times \frac{{f_{1} }}{{f_{2} }}.$$

Equation (3) clearly indicates that the ratio $x_{1} /x_{2}$ in the presence of resonators is different from the ratio without resonance (i.e., $x_{1} /x_{2} \approx f_{1} /f_{2}$ ), suggesting the enhancement of the amplitude ($\alpha$) and phase ($\beta$) owing to the coupled resonance. In Fig. 2e and f, we show the maximized ratio of acoustic pressures in two cavities $\max \left( {\left| {x_{2\left( 3 \right)} /x_{1} } \right|} \right)$ and the corresponding phase difference ($\angle \left[ {x_{2\left( 3 \right)} /x_{1} } \right]$) for frequencies ranging from 800 to 1300 Hz. The maximum values are determined considering all possible $\theta_{inc}$ within 0––350°. Near 900 Hz, peaks are seen due to the resonance. Additionally, both $\max \left( {\left| {x_{2\left( 3 \right)} /x_{1} } \right|} \right)$ and the phase increase with the frequency for the resonator-based sensor, which evidently surpass those of the reference cases [dashed curves in Fig. 2d] and implies that the contrast in measured signals, in terms of both amplitudes and phases, between the microphones increases for higher frequencies. The symbols capture the trend of the simulation results and the mismatch may again be attributed to the fabrication uncertainty and the noise stemming from the measurement environment.

### CNN performance on single-frequency sources

**Figure 3:**
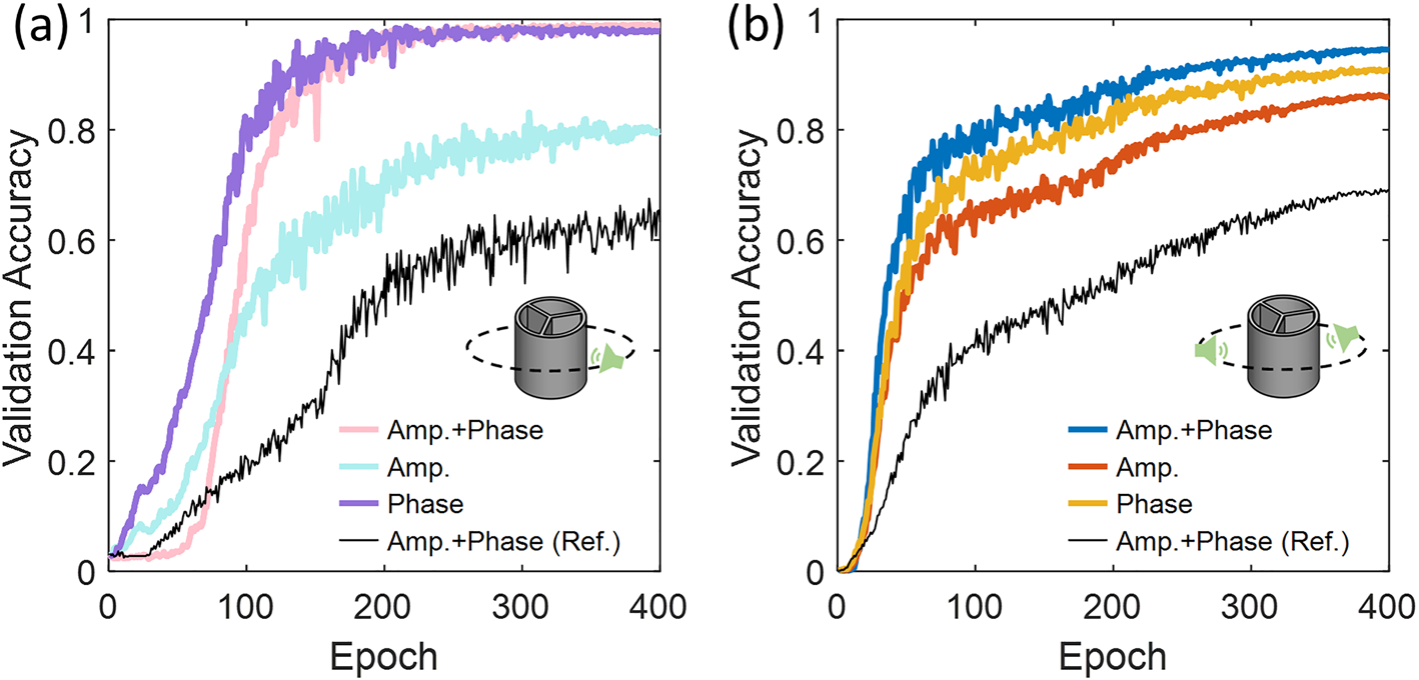
Validation accuracy of the CNNs trained using both amplitude and phase features, or either amplitude or phase feature on the prediction of the incident angle of (**a**) one single-frequency source and (**b**) two simultaneously active single-frequency sources. The black curves show the validation accuracy corresponding to the reference sensor trained by using both features.

Since the enhancement effect is most effective near the resonance, we start with training the CNN using the data collected from single-frequency signals over 800–1300 Hz (i.e., training used all single-frequency data in the range, while the prediction was made at an individual frequency within the range). We have separately trained three CNNs based on either amplitude or phase feature or both. As shown in Fig. 3a, after training the CNN for 400 epochs, the validation accuracy on predicting the incident angle for the network trained with both amplitude and phase features reached 98.9%, which slightly exceeds that trained by the phase feature (97.8%), while far outscores the accuracy achieved by the one trained based on the amplitude feature (only 79.7%). Notably, using phase alone appears to be better than using both amplitude and phase when epoch is less than 250; when the epoch is less than 100, the case of using both is slightly better than using phase alone. This is further confirmed by comparing Fig. S5b and S5c of the Supplementary Materials, where between 800 and 1100 Hz, using phase alone is slightly better than using both. Since the enhancement of the amplitude is strong near the resonance, the validation accuracy is expected to be poorer away the resonant frequency [see Fig. S5a in Section S5 of the Supplementary Materials]. Interestingly, for the phase feature, lower frequencies still provide enough differentiability to increase the validation accuracy, whereas reduced accuracy is observed much beyond the resonance, which may be somehow against the intuition, however, corroborates the simulation results that the resonators do not work as filters [see Fig. S5b in Section S5 of the Supplementary Materials]. When two features are used during the training, the validation accuracy is improved both below and beyond the resonant frequency, indicating these features can complement each other and create a synergy to enhance the sensing capability, seemingly suggests some interferences between the two features due to the resonators [see Fig. S5c in Section S5 of the Supplementary Materials]. It is noteworthy that networks trained by both features and the phase feature lead to comparable accuracies beyond ~ 200 epochs, yet training two features requires the minimization of both loss functions, which might have caused more epochs than training either feature to reach similar accuracies. We also remark that even though resonance effects are most prominent near the resonant frequency, they are still present across a broader range of frequencies, such as 700–1200 Hz, as demonstrated in Fig. 2b, e, and f. Consequently, the resonators contribute to maintaining a satisfactory validation accuracy even for frequencies below its resonant frequency, ensuring the effectiveness of the system across a wider frequency spectrum. We provide additional results in Section S6 of the Supplementary Materials when the network was trained by using only the data at one frequency within 800–1300 Hz and the prediction was made at the same frequency. We have also plotted the validation accuracy of the network trained by both features (best among three training scenarios) extracted from the same signals measured instead by reference sensor without resonators. The accuracy at 400 epochs arrives at 63.2% [see Fig. S7a in the Supplementary Materials], which is much lower than 98.9% of the resonator-based sensor. This suggests that the resonance effect refines the acoustic features and thus improves the CNN performance. On the test datasets, the CNNs trained by amplitude, phase, and both features using the signals collected on the resonator-based sensor read 67.6%, 96.1%, and 96.7%, respectively (see corresponding confusion matrix plots in Fig. S7c–S7e in the Supplementary Materials).

Next, we consider the case where two sources are simultaneously active. We narrow the frequency of the selected samples to 900–940 Hz to be closer to the designed resonance of the sensor. In fact, as long as the chosen range is closed to the resonance, the accuracy of angle prediction is insignificantly affected (see Fig. S8a of the Supplementary Materials). With $n = 3$, the combination ${\text{C}}\left( {36n,2} \right)$ yields 5778 possibilities and with 4 sets of measurement data we obtain 23112 samples for the CNN. As illustrated in Fig. 3b, compared with single source case (Fig. 3a), at 400 epochs, the validation accuracy of the network trained by both features arrives at 94.5%, which is better than those trained with either amplitude or phase feature by 3.7% and 8.4%. Again, the accuracy of the network trained by the phase feature is slightly better than that trained by the amplitude, which is similar to the single-frequency case in Fig. 3a. Further comparison between Fig. 3a and b indicates that the amplitude feature works better near the resonant frequency. We also note that, for the two-source case, the accuracy of the networks trained by both features and the phase feature slightly decreases than the single source case, implying that as the number of sources increases the sensing becomes more challenging. Additionally, it is expected that the spatial and spectral difference between two sources will impact the CNN accuracy as these factors influence the features used by the CNN to differentiate the sources. The accuracy is mostly improved when source frequencies are closer to the resonant frequency of the sensor. Improvement is also achieved for two sources having larger frequency difference. We provide additional results and related discussion in Section S8 of the Supplementary Materials. On test dataset, the networks achieve 80.2%, 86.9%, and 93.9% accuracies with amplitude, phase, and both features used for training, respectively (corresponding confusion matrix plots can be found in Fig. S9 in the Supplementary Materials). Again, the performance of CNNs trained by the data obtained from resonator-based sensor is compared against that trained using reference sensor data. Consistent with Fig. 3a observation, validation accuracy of the network trained by both features processed from reference sensor substantially decreases, corroborating the argument that resonance effect provides the CNN more effective acoustic features to make more accurate predictions. This is indeed observed in Fig. 2f, where an increased phase difference around 2 is seen near 900 Hz in the presence of resonators, which doubles that of the reference case in the absence of the resonators. The reference sensor in fact does not provide sufficiently useful phase features for the CNN to work with, as supported by the validation accuracy in Fig. S7a of the Supplementary Materials, which is clearly different from that of the resonator sensor (Fig. 3a). Additionally, we remark that the significance of the phase difference is expected to reduce as the device miniaturizes due to decreasing spatial separation between detectors. In such scenario, the importance of the amplitude variation preserved and modified by the resonators can be increasingly meaningful to train the CNN.

### CNN performance on siren sources

**Figure 4:**
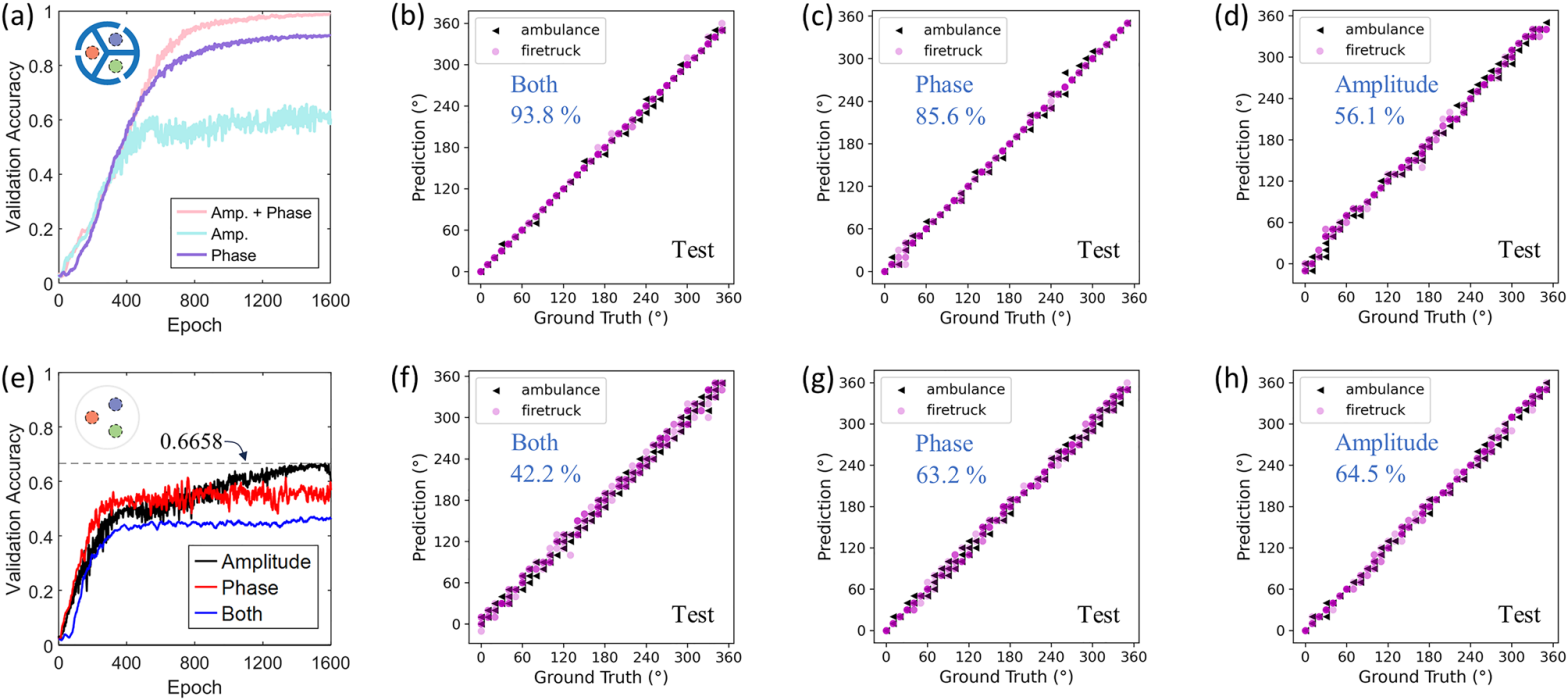
(**a**) Validation accuracies of CNNs trained by amplitude, phase, and both features extracted from siren signals measured by resonator-based sensor. (**b**)–(**d**) confusion matrix plots that quantify the performance of corresponding CNNs in (**a**) on test data (accuracy values shown by the blue font). (**e**) validation accuracies the CNNs trained by amplitude, phase, and both features extracted from siren signals measured by reference sensor (no resonators). (**f**)–(**h**) confusion matrix plots and accuracies of CNN on test data corresponding to the three scenarios in (**e**).

Figure 3 demonstrates that CNN can use the amplitude and phase features modified by the resonators more effectively to enhance the angle sensing capability for single-frequency signals. Next, we examine the acoustic sensing performance on siren sources. Figure 4a shows the validation accuracies of CNNs trained by different type of features. Specifically, the CNN trained by both features achieves the best accuracy on the validation dataset, reaching 98.6% after 1600 epochs of training. Notably, when trained with only the phase feature, the CNN’s performance on the validation set drops to 90.9% after 1600 epochs. When only the amplitude feature is utilized in the training, the validation accuracy significantly reduces to 60%. The comparison obviously indicates that CNN is most effective in predicting the incident angle of sirens when employing both amplitude and phase features. In Fig. 4b–d, the trained CNNs are further checked on testing data unseen during the training and validation. Consistent with Fig. 4a, when trained by both features (Fig. 4b), the predicted incident angle mostly falls within the range of ± 10° from the ground truths, achieving a test accuracy of 93.8%; when trained by either the phase (Fig. 4c) or the amplitude (Fig. 4d) feature, the test accuracies become 85.6% and 56.1%, respectively. The predicted incident angles deviate more from the ground truths and the CNNs produce more predictions with slightly large errors. We do not observe notable preference of better predicting either siren type from Fig. 4b–d; all three trained networks predict the siren type by 100% accuracy. The performance on the testing set again confirms the benefit of training the CNN with both features for enhanced acoustic sensing of sirens. Compared to Fig. 3a, in which single-frequency signals at 800 Hz–1300 Hz are employed, in Fig. 4, the siren signals themselves cover a much broader range of frequency, not to mention additional frequency components introduced by the background noise [see Fig. S3b–S3d in Section S4 of the Supplementary Materials for more details on the noise analysis]. By combining the resonator-based acoustic sensor and deep learning, the advantage of resonance effect which intuitively is expected to work for narrowband signals instead enable good sensing performance for broadband signals. To further demonstrate the benefit of the resonance effect, in Fig. 4e, we show the validation accuracies of the CNNs trained by amplitude, phase, and both feature extracted from siren signals [same ones used in Fig. 4a–d] measured by reference sensor without resonators. In sharp contrast to Fig. 4a, the CNNs performances are significantly poorer on validation data. Specifically, when trained only by amplitude, the CNN accuracy reaches 66.6%, while a reduced accuracy of 59.7% is obtained when trained by phase. This indicates that the reference sensor cannot provide sufficiently useful features for networks to achieve high accuracy as the resonator-based sensor. The network trained by both features has the worst validation accuracy of only 45.6%, which contradicts the trend observed in Fig. 4a. This suggests that, without resonators in the sensor, the two features negatively affect each other such that when used together the CNN accuracy is further impaired. These trained networks are subsequently evaluated on test data. In Fig. 4f–h, we can see that the network trained with both features realizes only 42.2% accuracy, followed by the ones trained with either phase or amplitude reaching 63.2% and 64.5%, respectively. The reductions are beyond 50% and 20% when comparing Fig. 4b with 4f and Fig. 4c with 4g. Despite an exception observed in Fig. 4h, where the reference sensor marginally outperforms the resonator-based sensor (both accuracies are comparably low), the side-by-side comparison in Fig. 4 clearly show the benefit of having resonators in the sensor to be more effectively paired with deep learning for improving the estimation of sound source direction. In the Section S10 of the Supplementary Materials, we provide the time-domain signal of representative ambulance and firetruck siren signals and their corresponding spectrograms. To ensure the data is not biased by the repeated measurement of siren signals, the training, validation, and test datasets are produced by alternative means. In one means, samples belonging to a specific recording, i.e., 1 to 6, are used for testing, while the remaining are utilized for training and validation. In the other, samples associated to a specific incident angle, i.e., 0 to 350° by every 10°, are chosen as the test data and the rest as the training and validating data for the CNN. As illustrated in Figs. S11 and S12 in the Supplementary Materials, test results of the CNNs trained by both means do not show remarkable difference depending on the way the data was divided (i.e., by the recording or by the incident angle), suggesting that the data does not contain bias and none of the improvement in the directional sensing is expected to arise from biased data.

## Conclusions

We have shown that after passing through a compact resonator-based acoustic sensor, both amplitude and phase of the incoming sound are enhanced near the resonant frequency and this acoustic information is not filtered by the resonators at off-resonance frequencies. Based on the resonance effect, convolutional neural networks have been trained using either amplitude or phase, or both features. When single-frequency signals are used, the network trained by both features demonstrates the best accuracy on predicting the angle of incident of either one or two sources. When siren signals with various background noises are utilized, the network consistently shows the highest accuracies on predicting the incident angle and the type of a single siren source despite the signals are broadband. Through comparison with the performance of CNNs trained with data collected on reference sensor without resonators which consistently show much lower accuracies, our results indicate the synergy between the resonance effect and deep learning for improving the performance of acoustic sensors, especially when designed in compact sizes, under which condition that the phase difference between detectors becomes increasingly difficult to be distinguished using conventional approaches. We hope that these results will help to pave the way for the development of compact, high-accuracy acoustic sensors for various applications including detection of emergency vehicles in autonomous driving systems.

### Supplementary Information

Supplementary Information.

## Data Availability

The data used in this study is available from the corresponding authors upon reasonable request.
