# Supplementary material for: Robust acoustic directional sensing enabled by synergy between resonator-based sensor and deep learning
Source: Sci Rep. 2024 May 2;14:10148. doi: 10.1038/s41598-024-60696-1 (PMC11066120; doi:10.1038/s41598-024-60696-1)
Supplement: Supplementary file 1 — Supplementary Information. [file 41598_2024_60696_MOESM1_ESM.docx]

**Supplementary Materials: Robust Acoustic Directional Sensing Enabled by Synergy between Resonator-based Sensor and Deep Learning**

Ziqi Yu^a^, Xiaopeng Li^a^, Hojung Jung^b^, Masahiro Harada^b^, Danil Prokhorov^a^, Taehwa Lee^a^

^a^Toyota Research Institute of North America, Toyota Motor North America, Ann Arbor, Michigan, 48105, USA

^b^Toyota Motor Corporation, 1200 Mishuku, Susono, Shizuoka 410-1107, Japan

**S1. Experimental setup and design of acoustic sensor**

The experimental setup used for measuring the response of our acoustic sensor is shown in Fig. S1(a). A loudspeaker is used to play the recorded siren signals. The response of the acoustic sensor is measured in a room without any acoustic treatment. Different incident angles are realized by a motorized rotation stage (as shown in the photo). Care has been taken when adjusting the alignment between the loudspeaker and the sensor. Fig. S1(b) illustrates the design of the acoustic sensor where three cavities exhibiting a C_3_ symmetry is separated by inner walls of *t* = 2 mm thick; the outer wall thickness and the sensor’s height are *t* = 2 mm and *h* = 25 mm, respectively. The diameter of the sensor is *d* = 26 mm, and the height and width of the slit are *w*_s_ = 1 mm and *h*_s_ = 10 mm. Photos also depict the fabricated sensors with surface microphones inserted. The diameter and the thickness of these microphones are *D*­_mic_ = 13.2 mm and *t*_mic_ = 3.2 mm, respectively.

Fig. S1 (a) Schematic and pictorial illustrations of the measurement environment and setup. (b) Schematic of the fabricated sub-wavelength acoustic sensor with the surface microphones mounted (zoom-in view shows the dimensions of the surface microphone).

**S2. Details about convolutional neural network**

Detailed architecture of the CNN used in the main text is provided in Fig. S2. We note that the following CNN is used for the prediction of siren sources, where the output is separated into two layers with one predicting the incident angle of the siren while the other predicting the siren type. For single-frequency cases, slight modifications are implemented at the CNN output to a single layer that predicts the incident angle of either one (for single-source case, as in FIG. 3(a) of main text) or two (for two-source case, as in FIG. 3(b) of main text). The layers of consisting of “Conv2D”, “Max-pooling”, and “Dropout” are repeated four times (4x), where the dropout rate is kept as 0.2, whereas the number of filters in the Conv2D layers are 8, 16, 32, and 32 with the respective kernel size of (7,7), (5,5), (3,3), and (3,3) and the pooling size in the Max-pooling layers is kept as (2,2).


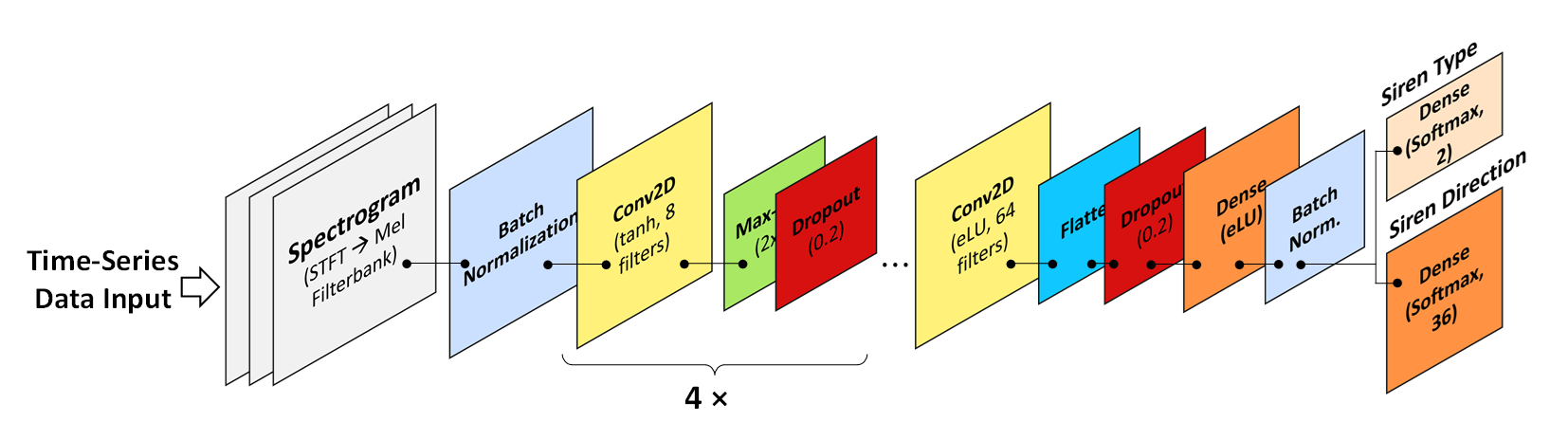


Fig. S2. Schematic illustration of the CNN for predicting the incident angle and the type of sirens.

**S3. Noise analysis of siren signals**

In Fig. S3, we provide noise analysis of the recorded siren signals used in our experiments. For single-frequency source, We have plotted the measured spectrum for a single frequency measurement, which shows measurement noise; clearly, the spectrum exhibits a noise floor that is 30 dB lower than that of the excitation frequency (i.e., 1300 Hz). For siren measurement, the used siren source [Kaggle open-source data, available at [1]; Fig. S3(c)] is a lot noisier than the clean siren [open-source data, available at [2]; Fig. S3(b)]. Moreover, the detected spectrum contains further noise from our measurement, as shown in Fig. S3(c). Particularly, the noise floor increase.


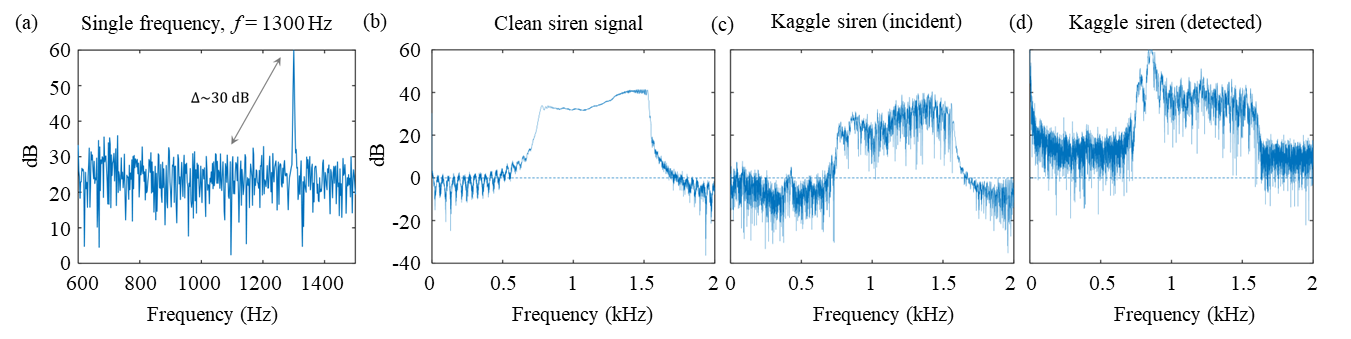


Fig. S3 (a) Single frequency measurement showing a noise level of 30 dB (quiet room). Siren measurement showing noise. The spectra for the (b) clean siren [2], (c) Kaggle siren [1] used as the incident in the measurement, and (d) detected Kaggle siren in the measurement.

**S4. Damping coefficients in the resonators model**

Damping coefficients $\gamma$, $\gamma_{0}$, and $\delta$ used in Eq. (2) and Eq. (3) of the main text are numerically extracted from COMSOL model. Their dependence on the frequency is illustrated in Fig. S4(a). Next, we show the comparison between the numerically obtained and analytically calculated sound pressure spectra for different $\delta$ values and number of resonators in the sensor. When only one cavity houses a resonator, as shown in Fig. S4(b) and S4(c), both $\delta=0$ and $\delta=0.1\omega_{0}$ illustrate good agreement between the results from COMSOL and analytical model. The analytical expression used in this calculation reads $\left| p \right|=\left( p_{A}\frac{\gamma_{s}S}{V} \right)\left| \frac{f_{ext}/m}{\omega^{2}-\omega_{0}^{2}+Im\left( \gamma\right)-i\left( \delta+Re\left( \gamma\right) \right)} \right|$. For three-resonator case, the results from two methods match as well, [Fig. S4 (d)], with analytical expression $\left| p_{1} \right|=\left( p_{A}\frac{\gamma_{s}S}{V} \right)\left| \frac{f_{ext,1}/m}{\omega^{2}-\omega_{0}^{2}+Im\left( \gamma+\gamma_{c}X_{21}+\gamma_{c}X_{31} \right)-i\left( \delta+Re\left( \gamma+\gamma_{c}X_{21}+\gamma_{c}X_{31} \right) \right)} \right|$.

Fig. S4 (a) Damping coefficients $\gamma$, $\gamma_{0}$, and $\delta$ extracted from numerical model. Acoustic pressure calculated in COMSOL and its comparison with analytical model for the acoustic sensor with a single resonator setting $\delta$ to (b) 0 and (c) $0.1\omega_{0}$. (d) Comparison of acoustic pressures for the three-resonator sensor obtained from COMSOL and analytical model.

**S5. Frequency-dependent validation accuracy on single-frequency sources**

In Fig. S5, we provide validation accuracies of three scenario. In Fig. S5(a), the CNN is trained utilizing only the amplitude feature. We observed reduced accuracy at frequencies below and beyond the resonance. In Fig. S5(b), the validation accuracy is significantly improved compared to Fig. S5(b), though passing 1200 Hz, the accuracy starts to decrease. In Fig. S5(c), when both amplitude and phase features are employed during the training, in comparison to Fig. S5(b), the accuracy beyond 1200 Hz is enhanced, which suggests a complementary effect due to the addition of the amplitude feature and clearly indicate the benefit of employing both features for improving the acoustic sensing.

Fig. S5. Validation accuracy of the CNN on predicting the incident angle of single-frequency sources when the network is trained using (a) the amplitude feature, (b) the phase feature, and (c) both features.

**S6. Validation accuracy on single-frequency sources with single-frequency training data**

In Fig. S6, we provide additional data of the single-source case in supplement to the results shown in FIG. 3 in the main text. Instead of training the CNN using the single-frequency source ranging in 800 – 1300 Hz and 900 – 940 Hz, as used for FIGs. 3(a) and 3(b), respectively, in the main text, in Fig. S6(a), we show the validation accuracy of the CNN predicting the incident angle of single source with a frequency chosen from 800 – 1300 Hz. Note that for each data point, the source frequency used in the training and validation are the same. Both amplitude and phase features are used during training. It can be seen that the validation accuracy peaks near 900 Hz, where the design resonant frequency of our acoustic sensor locates. Towards both ends, accuracy drops. These results suggest the important role played by the resonators in our sensors. In Fig. S6(b), the CNN is trained by single source with a frequency picked from a range having three consecutive frequencies. Similar to Fig. S6(a), the source frequencies used in the training and validation are drawn from the same range. As illustrated by the short lines in Fig. S6(b), the best accuracy again is achieved around the resonant frequency of 900 Hz, while for lower and higher frequencies, the accuracy decreases. Comparing the results in Figs. S6(a) and S6(b), it is seemingly true that when mixed-frequency sources are used in the training, the CNN tends to provide more robust prediction performance. This is because that the CNN is more capable of differentiating sources at differed frequencies, which improves the overall accuracy.


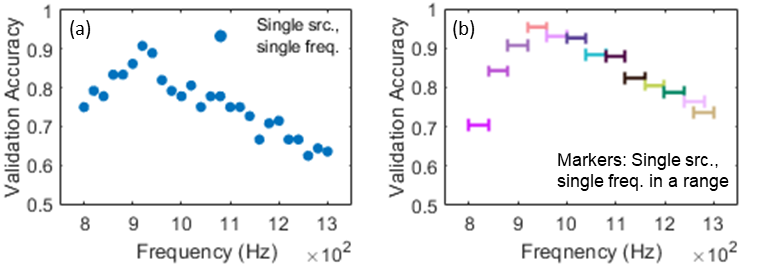


Fig. S6 Validation accuracy of the CNN trained by using experimentally measured response of the acoustic sensor to a single-frequency source. Only one source is used. In (a), each data point is trained by a single-frequency between 800 Hz and 1300 Hz (20 Hz interval). In (b), each line represents the validation accuracy of the CNN to a single-frequency source with the frequency in a range spanning three consecutive frequencies, i.e., $f_{1},f_{2},f_{3}\in\left[ f-\Delta f,f+\Delta f \right]\left. \right|_{\Delta f=20 \mathrm{Hz}}$. Note that the vertical bars do not represent error bars.

**S7. Test accuracy of CNNs on single-frequency sources (one active source, resonator-based sensor)**

Fig. S7(a) depicts the validation accuracy of the CNNs trained by amplitude, phase, and both features using the data measured on reference sensor, which all show much lower values at 400 epochs compared to those of resonator-based sensor [Fig. S7(b)]. The test results are skipped since the validation accuracies are suggestive of comparably low accuracies on the test dataset.

Fig. S7(c)-S7(e) show the test results and the confusion matrix plots that compare the CNN predictions against the ground truth for single-frequency sources at locating at different angles. The CNNs are trained by only amplitude [S7(c)], only phase [S7(d)], or both features [S7(e)].

Fig. S7 Test accuracies on single-frequency signals obtained from the CNNs trained by (c) amplitude, (d) phase, and (e) both features. Only one source is active. Note that results in Fig. S7(b) are also a part of FIG. 3(a) in the main text.

**S8. Analysis of validation accuracy in two-source case with varying source frequencies**

We consider the two-source scenarios where the frequencies of the sources were either chosen to be the same one (closest frequencies) or ones drawn from a range (close or far frequencies, and the same frequency allowed). If we denote the two frequencies $f_{1}$ and $f_{2}$ and the frequency range $\left[ f_{a},f_{b} \right]$. Then the same frequency case may be represented as $f_{1},f_{2}\in\left[ f_{a},f_{b} \right] \left. \right|_{f_{a}=f_{b}}$, whereas the different frequency case may be represented as $f_{1},f_{2}\in\left[ f_{a},f_{b} \right] \left. \right|_{f_{a}\neq f_{b}}$. For the training of the CNN, we considered using both amplitude and phase features.

In the main text, we use the reduced frequency range 900 – 940 Hz in the training for the two-source case. We provide additional figures to show that the validation accuracy of the CNN is not significantly affected by the selection of the reduced frequency range. The main motivation of using narrower frequency range is to take advantage of the enhancement effect near the resonant frequency.

In Fig. S8(a), we compare validation accuracies obtained by two different range of frequencies, with one being 900 – 940 Hz having three discrete values (900, 920, 940) Hz which is used in the main text FIG. 3(b), and one more being 880 – 920 Hz with three discrete values (880, 900, 920) Hz. When training the CNN, the frequencies of the two sources are drawn from the frequency range, allowing repetition of the same frequency. It is clearly observed that the difference between the two accuracies obtained from training the CNN using 900 – 940 Hz (green line) and 880 – 920 Hz (orange line) is negligible since they are both closed to the resonance. We note that the vertical bars on two ends of each marker indicates the end frequencies of the range. As the data is discrete, the line in-between the bars are not expected to show the frequency dependence and is only used for visualization purpose.

In Fig. S8(a), when three frequencies are used for the two sources, in addition to the two ranges (880, 900, 920) Hz and (900, 920, 940) Hz that are closed to the resonance at 900 Hz, we further studied a case when the three frequencies (1260, 1280, 1300) Hz which are far from the resonant frequency. As shown by the purple line in Fig. S8(a), the validation frequency drops by nearly 10 %, implying that the performance of the sensor degrades for off-resonance sources and such degradation is not compensated by the CNN which suggests the importance of resonator for our hybrid approach. When two frequencies are chosen for the sources, as illustrated by the magenta line [(800, 1300) Hz] and the light-blue line [(880, 900) Hz], validation accuracies are reduced compared to the three-frequency cases expect for (1260, 1280, 1300) Hz which greatly deviates from the resonant frequency. It is also observed that the accuracy is poorer when the two frequencies are away from the resonant frequency, which agrees with the observation of the three-frequency cases. Finally, we considered the case where the two sources are at the same frequency. The validation accuracy data is plotted for each frequency from 800 Hz to 1300 Hz. Despite the fluctuations, it can be roughly seen that the accuracy is higher near 900 Hz and generally tends to go down moving towards higher frequencies. All the single-frequency accuracies are much lower than the two-frequency and three-frequency cases. We note that the single-frequency scenario is equivalently the case when the two sources have the closest frequency. Our hybrid approach showed relatively poorer performance on the single-frequency scenario because different frequencies provide differentiability for the CNN.

Moreover, we show the validation accuracy as a function of the difference between the incident angles of the two sources in Fig. S8(b) – S8(d). The incident angle difference ranges from 10 ° to 350 °. We note that the angle difference increases between 0 ° to 180 °; beyond 180 °, the angle difference decreases spatially between the two source locations. Ideally, we expect the data to be symmetric with respect to 180 °. But it is not the case in Fig. S8(b) – S8(d) due to uneven distribution of data for the angle difference. We also note that for large angle difference, e.g., 320 ° to 350 °, the number of data is less compared to smaller angle difference and thus larger fluctuation of validation accuracy is observed. In Fig. S8(b), we can see that when the two sources are both at 900 Hz, the validation accuracy for 10 ° angle difference is around half and gradually increases as the angle difference becomes larger while showing slight decrease beyond 120 °. The data roughly depicts a symmetry about 180 °. In Fig. S8(b), for the two-frequency case, the validation accuracy for (880, 900) Hz case is higher than that of the (800, 1300) Hz case for most values of the angle difference, indicating the effect of the resonance. High accuracies mainly appear for larger angle difference around 180 °, whereas the accuracy quickly decay for very small angle differences between the two sources. Similar observation can be made for the three-frequency case, as depicted in Fig. S8(d).

Based on the results in Fig. S8(b) – S8(d), we can see that the training is successful in differentiating the two sources separated by the angle ranging from 10 ° to 350 °, with the accuracy peaking for larger spatial angle difference. Moreover, the accuracy is improved when the two sources are at different frequencies especially when the frequencies are closed to the resonant frequency of the sensor.


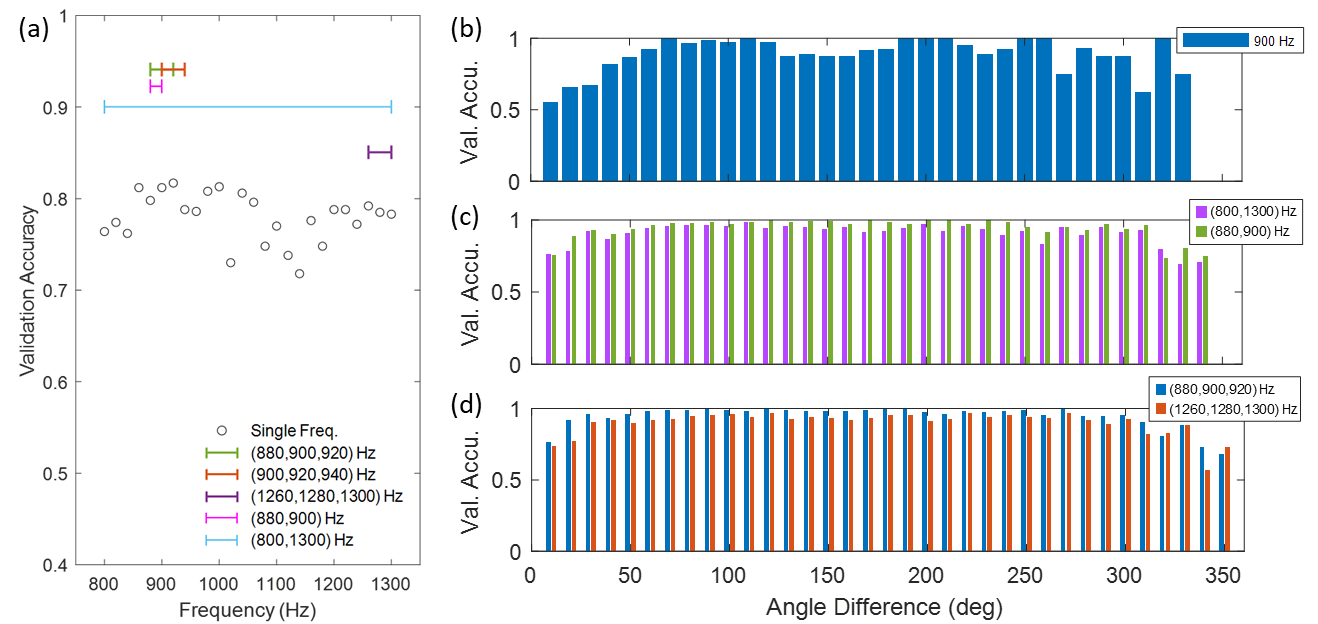


Fig. S8 (a) Validation accuracy for predicting incident angles of two sources. The frequencies of the sources can be the same (“Single freq” in the legend) or different (others in the legend, for example, (900, 920, 940) Hz means each source can be at one of the three values). Validation accuracy for the incident angle prediction of two sources as a function of incident angle difference with (b) two sources having the same frequency of 900 Hz, (c) two sources having two frequencies of either (800, 1300) Hz, or (880, 900) Hz, (d) two sources having three frequencies of either 800 Hz, 900 Hz, and 920 Hz, or 1260 Hz, 1280 Hz, and 1300 Hz.

**S9. Test accuracy of CNNs on single-frequency sources (two active sources, resonator-based sensor)**

As illustrated in Fig. S9, the test accuracy for the CNNs on two single-frequency sources are 80.2 %, 86.9 %, and 93.9 % when trained by amplitude, phase, and both features. Since for this case the sample population was much bigger (synthesized by picking two single-frequency signals and placing at two angles), the number of test samples was nearly 2000. In the following plots, a fair number of outliers deviating from the main diagonal can be seen. However, compared with those residing closer to the main diagonal, indicating more accurate predictions, the number of bad predictions is much less, and these facts can be properly reflected by the test accuracy values.


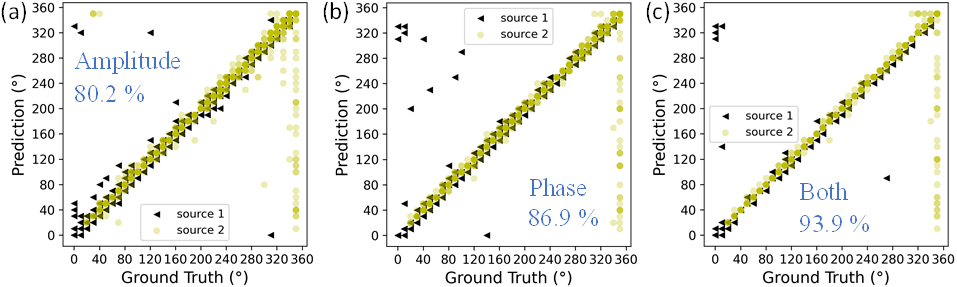


Fig. S9 Test accuracies of the CNNs trained on two single-frequency sources by (a) amplitude, (b) phase, and (c) both features. The data was collected by resonator-based sensor.

**S10. Responses of acoustic sensor on broadband siren signals**

In Fig. S10, we plot the time-series data of the ambulance and firetruck sirens and the corresponding spectrograms. We can see that the two sirens exhibit distinct characteristics in the time domain; their spectrograms show responses over broad spectra up to 6 kHz for the ambulance siren and 10 kHz for the firetruck siren, respectively, and the distribution of high-dB levels differ as well. These are expected to provide sufficient differentiability to the CNN to recognize their types.

On the other hand, superposing cavities supporting different resonant frequencies may enable broader bandwidth of our sensor. For the current design, with a designed resonant frequency at around 900 Hz, with CNN, our hybrid approach can predict the incident angle of the sirens at high accuracy, even if the siren signals cover much broader frequency range.


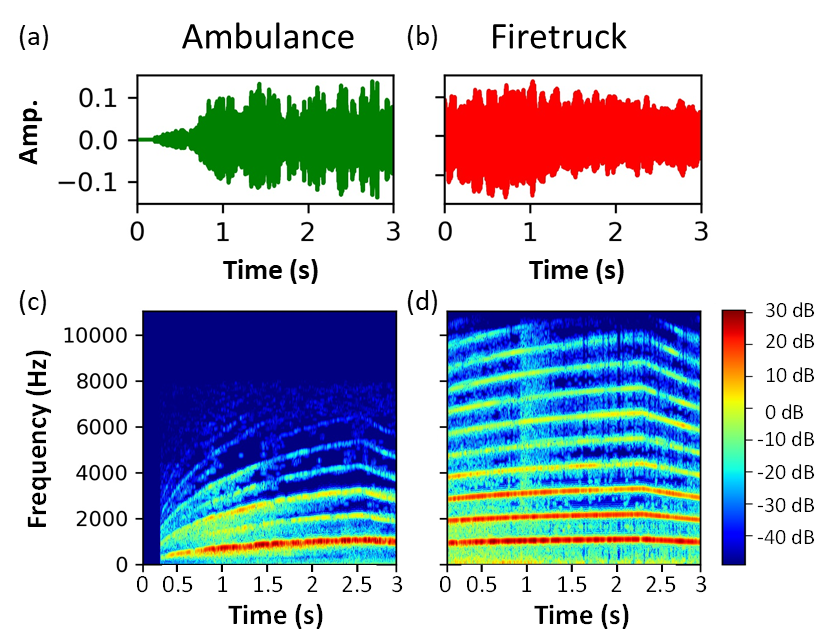


Fig. S10 Time-series signals of (a) ambulance and (b) firetruck sirens. Spectrograms of the (c) ambulance and (d) firetruck sirens.

**S11. The effect of data splitting on the CNN performance**

The siren samples used to train the CNN in the main text is obtained by measuring a siren signal 6 times (6 recordings) for varying incident angles for all siren signals. The samples are then dispersed into the training, validation, and testing datasets by a ratio. To check if repeated measurement introduces any bias into the data, in this section, the testing dataset is obtained alternatively.

First, we split the data such that the testing dataset only has samples from a specific recording. The remaining samples are subsequently split into the training and validation sets. By doing these steps, we obtain 6 testing datasets and correspondingly 6 sets of training/validation data. We then train 6 CNNs (using both amplitude and phase features) and the trained networks are tested by their corresponding testing datasets. In Figure S11, we show the testing results for the 6 CNNs. After being trained 1600 epochs, all the 6 networks reached a validation accuracy above 90%. The plots appear different as expected due to differences among recordings. The test accuracies all exceed 90% (ranging from 94.9 % to 99.4 %) and we do not observe remarkable discrepancy. This suggests that the CNN training is not strongly affected by the recording, which implies that the data does not introduce noticeable bias.

Figure S11 Test results of the trained CNNs using the test dataset selected based on the recording number.

Second, we train the CNNs by taking the samples corresponding to the j^th^ incident angle, where j = 1, 2, …, 36, as the test data and the remaining as the training/validation data. We select angles of 0°, 30°, 60°, 70°, 90°, 120°, 150°, 180°, 190°, 210°, 240°, 270°, 280°, 300°, 330°, and 350°. The angles of 70°, 190°, 280°, and 350° are included to check the existence of potential effect introduced by an angle that is a multiple of 30°. The test results for these CNNs are provided in Figure S12. Among all the tests, the trained CNNs consistently predict the two angles that are closest to the test angle, the one which has been removed from the training/validation. For example, when the test angle is 0°, the network predicts 10° or 350°, and when the test angle is 70°, the CNN predictions are 60° or 80°. Since our CNNs perform classification, the trained CNNs map the unseen signals and their corresponding features to the closest resemblances learnt during the training, i.e., the two closest neighbors the test angle. Because we have chosen an angle interval of 10° in the measurement, the test results suggest an uncertainty within ±10°. We expect that using a regressor will allow more accurate prediction of the angle that is unseen in the training due to the angle interval used in the experiment. In addition, no specific test angles appear outstanding, which suggests that the data does not contain bias associated with the incident angle.


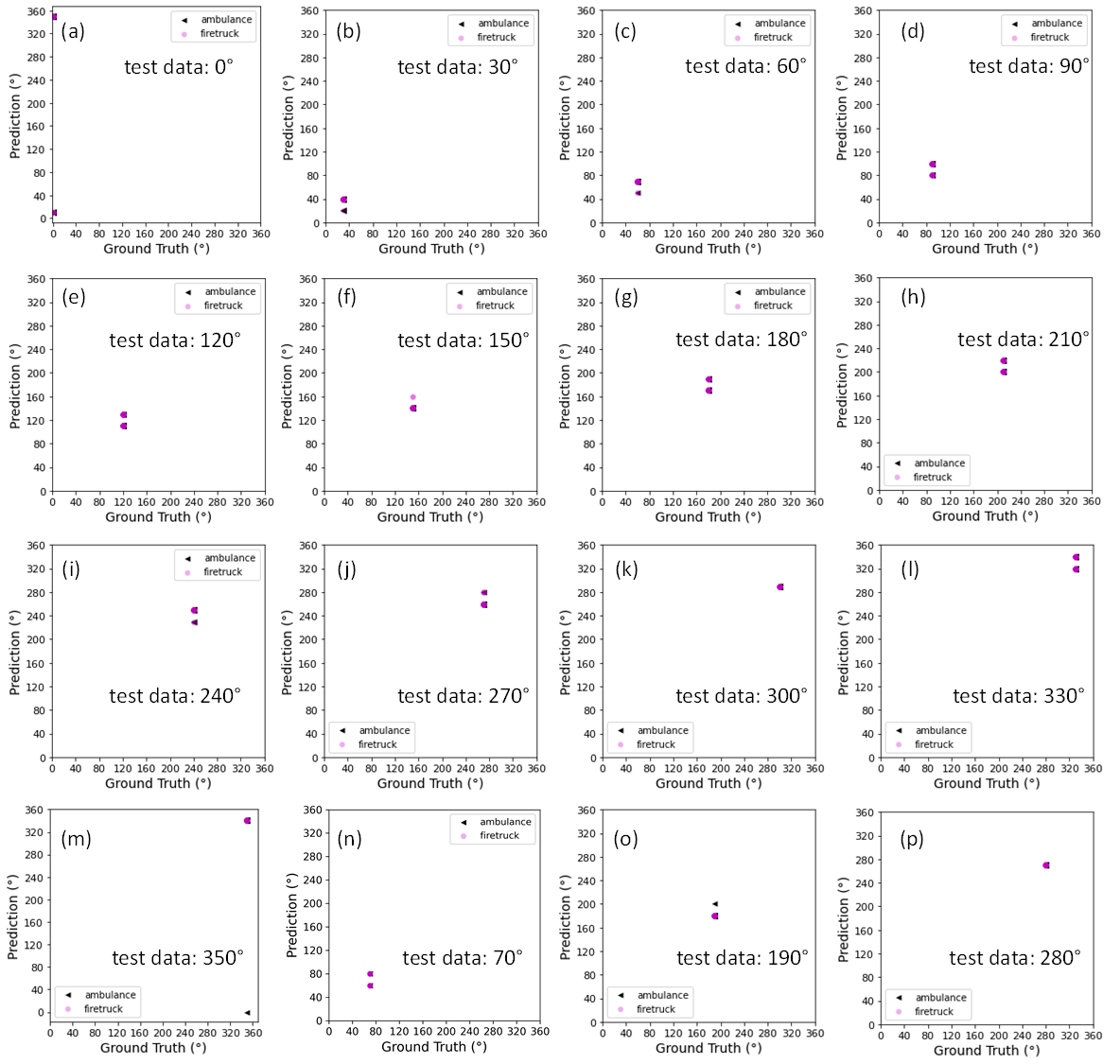


Figure S12 Test results of the CNNs by using the test data associated with a specific incident angle while using the remaining data for training/validation.

References

[1] https://www.kaggle.com/datasets/vishnu0399/emergency-vehicle-siren-sounds, (unpublished).

[2] https://www.galls.com/pages/siren-tones, (unpublished).
